# Triglyceride glucose-body mass index and cardiovascular mortality in patients undergoing peritoneal dialysis: a retrospective cohort study

**DOI:** 10.1186/s12944-023-01892-2

**Published:** 2023-09-05

**Authors:** Cuixia Zhan, Yuan Peng, Hongjian Ye, Xiangwen Diao, Chunyan Yi, Qunying Guo, Wei Chen, Xiao Yang

**Affiliations:** 1 Department of Nephrology, The First Affiliated Hospital, Sun Yat-sen University, 58th, Zhongshan Road II, Guangzhou, 510080 China; 2 NHC Key Laboratory of Clinical Nephrology, Guangdong Provincial Key Laboratory of Nephrology, Sun Yat-Sen University, Guangzhou, 510080 China; 3 Department of Nephrology, Ganzhou People’s Hospital (The Affiliated Ganzhou Hospital of Nanchang University), Ganzhou, 341000 China

**Keywords:** TyG-BMI, Cardiovascular mortality, Peritoneal dialysis

## Abstract

**Background:**

Recent studies have shown that triglyceride glucose-body mass index (TyG-BMI) is associated with the risk of ischemic stroke and coronary artery disease. However, little attention has been given to the association between TyG-BMI and cardiovascular disease (CVD) mortality in patients undergoing peritoneal dialysis (PD). Therefore, this study aimed to explore the relationship between TyG-BMI and CVD mortality in southern Chinese patients undergoing PD.

**Methods:**

Incident patients receiving PD from January 1, 2006, to December 31, 2018, with baseline serum triglyceride, glucose, and body mass index (BMI) information, were recruited for this single-center retrospective cohort study. TyG-BMI was calculated based on fasting plasma glucose, triglyceride, and BMI values. The association between TyG-BMI, CVD and all-cause mortality was evaluated using a multivariate-adjusted Cox proportional hazard regression model.

**Results:**

Of 2,335 patients, the mean age was 46.1 ± 14.8 years; 1,382 (59.2%) were male, and 564 (24.2%) had diabetes. The median TyG-BMI was 183.7 (165.5–209.2). Multivariate linear regression showed that advanced age, male sex, history of CVD, higher levels of albumin and low-density lipoprotein cholesterol, and higher urine output were correlated with a higher TyG-BMI (*P* < 0.05). During a median follow-up period of 46.6 (22.4–78.0) months, 615 patients died, of whom 297 (48.2%) died as a result of CVD. After adjusting for demographics and comorbidities, TyG-BMI was significantly associated with an increased risk of CVD mortality (hazard ratio [HR] 1.51, 95% confidence interval [CI] 1.05–2.17) and all-cause mortality (HR 1.36, 95% CI 1.05–1.75). After full adjustment, the 28% risk of CVD mortality (HR 1.28, 95% CI 1.13–1.45) and 19% risk of all-cause mortality were elevated (HR 1.19, 95% CI 1.09–1.31) when TyG-BMI increased by 1 stand deviation (SD) (34.2).

**Conclusions:**

A higher baseline TyG-BMI was independently associated with an increased risk of CVD and all-cause mortality in patients receiving PD.

**Supplementary Information:**

The online version contains supplementary material available at 10.1186/s12944-023-01892-2.

## Background

Cardiovascular disease (CVD) accounts for 52.7% of deaths in the peritoneal dialysis (PD) population [1]. In addition to classic factors (dyslipidemia, diabetes, and obesity) and chronic kidney disease (CKD)-related factors (inflammation, malnutrition, and fluid overload), PD-specific factors (peritoneal glucose exposure, advanced glycation end products, and bioincompatible solutions) play pivotal roles in the increased risk of CVD in dialysis patients [2, 3]. Despite substantial advances in the treatment and management of CVD in the PD population in recent years, mortality remains high, suggesting that there are huge obstacles that need to be overcome to prevent or delay the development of CVD [4–6].

A composite measure of plasma triglyceride (TG), fasting blood glucose (FBG), and body mass index (BMI), TyG-BMI, has recently been identified as a substitute target for insulin resistance (IR) [7–9], which has been identified as a risk factor associated with CVD [10]. The index simultaneously combines glucose, lipid levels and BMI, reflecting multiple critical factors in the management of PD. Recent studies have shown that TyG-BMI was associated with the risk of ischemic stroke, hypertension, and coronary artery disease [11–13]. However, its relationship with CVD mortality in the PD population remains unclear. Therefore, this study aimed to explore the association of TyG-BMI with CVD mortality based on a large-cohort Chinese PD population.

## Methods

### Study design and participants

This retrospective cohort study was conducted at a large PD center in southern China. From January 1, 2006, to December 31, 2018, end-stage renal disease (ESRD) patients who were catheterized and followed up at our PD center were recruited. Patients under the age of 18 years at the onset of PD treatment and those who had malignant tumors, suspended PD treatment within 3 months, had a failed kidney transplantation or were transferred from hemodialysis were excluded from the study. The project was approved by the Human Ethics Committee of the First Affiliated Hospital of Sun Yat-sen University.

### Data collection

Information on demographics and relevant disease conditions during patient admission to the PD center was collected. Demographic characteristics included age, sex, primary cause of ESRD, and comorbidities (diabetes mellitus, history of CVD, and hypertension). The participants were considered to have diabetes mellitus if they were diagnosed with diabetes by an endocrinologist or were taking antidiabetic medication. Participants with any of the following events were considered to have CVD: coronary artery bypass grafting, myocardial infarction, heart failure, angioplasty, stroke, or angina pectoris [14]. Blood pressure levels that exceeded 140/90 mmHg after repeated measurements in a rest state or hypotensive drug use were defined as hypertension.

Laboratory parameters were acquired during the initial three months of PD treatment. Fasting blood samples were collected and analyzed in the hospital laboratory. Levels of hemoglobin, serum albumin, FBG, total cholesterol (TC), TG, low-density lipoprotein cholesterol (LDL-C), high-density lipoprotein cholesterol (HDL-C), serum urea nitrogen, serum creatinine, uric acid, and high-sensitivity C-reactive protein (hs-CRP) were assessed. The estimated glomerular filtration rate (eGFR) was calculated with the CKD epidemiology collaboration formula. BMI, systolic blood pressure (SBP), diastolic blood pressure (DBP), and urine output were measured synchronously. Medication history, including the use of antihypertensive agents, hypoglycemic agents, and lipid-lowering medications, was recorded.

The equation for calculating the index is as follows:

TyG = Ln[(1/2FBG(mg/dL))×TG(mg/dL)] [15],

BMI = weight divided by height^2^,

TyG-BMI = TyG×BMI [7].

### Study outcomes and definition

The primary and secondary end points were CVD and all-cause mortality, respectively. The criteria for CVD death were death attributed to cerebrovascular disorders, anoxic encephalopathy, peripheral arterial disease, ischemic brain injury, congestive heart failure, cardiomyopathy, cardiac arrhythmia, cardiac arrest, acute myocardial infarction, or atherosclerotic heart disease [14]. The cause of death was identified by the comprehensive management team of the PD center, which consisted of primary and senior professors.

All patients were followed up until death, conversion to hemodialysis therapy, receipt of a kidney transplant, transfer to another center, loss to follow-up, or December 31, 2021.

### Statistical analysis

According to quartiles (Q) of TyG-BMI levels, participants were divided into four groups: Q1, < 165.5; Q2, 165.5-<183.7; Q3, 183.7-<209.2; and Q4, > 209.2. The results are reported as frequencies and percentages for categorical data, means and standard deviations for normally distributed data, and medians and interquartile ranges for nonnormally distributed data. Differences between groups were compared by Student’s t test, Mann–Whitney U test or *χ*^*2*^ test.

A linear regression model was used to analyze the relevant parameters of TyG-BMI. Kaplan–Meier curves were plotted to analyze survival time, and the distributions of survival among TyG-BMI quartiles were assessed by a log-rank test. The association between TyG-BMI and CVD and all-cause mortality was examined in Cox proportional hazards models. TG, FBG, and BMI comprise the TyG-BMI, which is closely related to diabetes. Therefore, the adjusted model did not include diabetes to avoid apparent bias caused by the correcting variable. The results are shown as hazard ratios (HRs) and 95% confidence intervals (CIs). To further explore the association between TyG-BMI and CVD and all-cause death, subgroup analyses were performed using clinical parameters, and the results are shown in a forest plot. All statistical analyses were performed using SPSS software version 26.0 (IBM Corp., Armonk, NY, USA); a value of *P* < 0.05 was considered as statistically significant.

## Results

### Baseline characteristics of participants

**Fig. 1:**
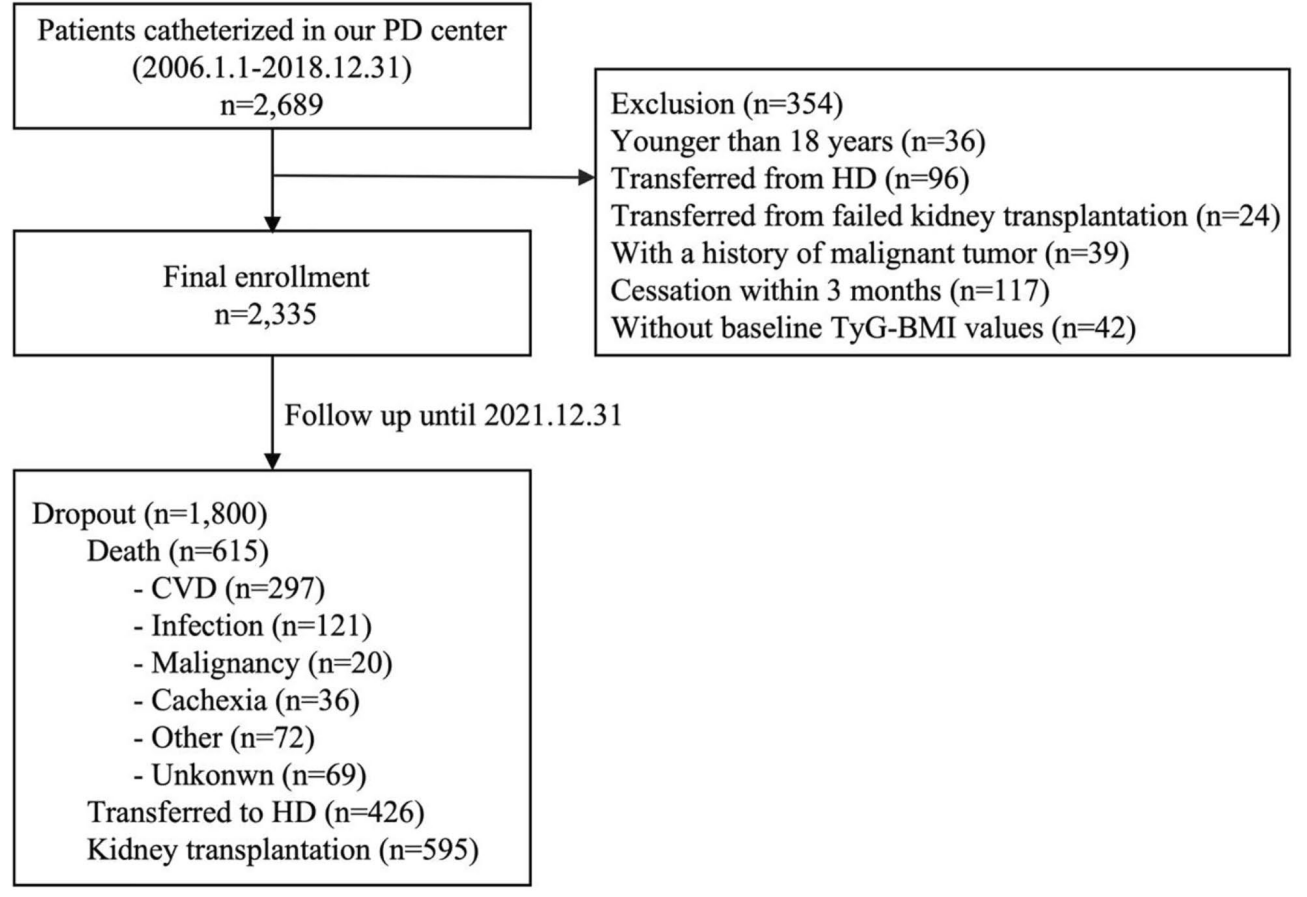
Follow chart for study participant enrollment and outcomes Note: PD peritoneal dialysis, HD hemodialysis, CVD cardiovascular disease

In total, 2,689 patients undergoing PD who were catheterized at our PD center were recruited. Of these, 354 patients who were aged < 18 years, transferred from maintenance hemodialysis, underwent failed kidney transplantation, had malignant tumors, received PD for less than 3 months, or lacked baseline TyG-BMI data were excluded. The remaining 2,335 participants were eligible for the final analysis (Fig. 1).

Participants’ mean age was 46.1 ± 14.8 years; 1,382 (59.2%) were male, and diabetic patients accounted for 24.2% (n = 564). The primary renal diseases were chronic glomerulonephritis (61.3%), diabetic nephropathy (20.6%) and hypertension (7.7%). Baseline TyG-BMI ranged from 82.0 to 380.0 (median 183.7, interquartile range 165.5–209.2). The baseline characteristics of the participants by quartiles of TyG-BMI are presented in Table 1. Compared with patients in Q1, patients with higher TyG-BMI levels were older; had a higher incidence of diabetes, CVD, and hypertension; had a higher proportion of males; and had increased urine output and higher TC, LDL-C, uric acid, and hs-CRP levels but lower DBP and HDL-C levels (P < 0.05) (Table 1). There was no obvious distinction among the groups regarding SBP, hemoglobin, albumin, serum creatinine, urea nitrogen, and eGFR levels (P > 0.05) (Table 1).

**Table 1:** Baseline characteristics of different TyG-BMI groups

Characteristics	Total (n = 2,335)	Quartiles of TyG-BMI					*P* value
		Q1 (n = 584) < 165.5	Q2 (n = 584) 165.5-<183.7		Q3 (n = 584) 183.7-<209.2	Q4 (n = 583) > 209.2	
Age (years)	46.1 ± 14.8	40.4 ± 15.1	45.2 ± 15.1		46.7 ± 13.8	50.2 ± 13.3	< 0.001*
Male sex (n, %)	1382 (59.2)	296 (50.7)	357 (61.1)		376 (64.4)	353 (60.5)	< 0.001*
Dialysis duration (months)	46.6 (22.4–78.0)	48.8 (21.8–89.0)	46.0 (21.6–76.2)		47.9 (24.4–75.3)	43.5 (21.9–70.7)	0.076
Primary renal diseases							
Glomerulonephritis (n, %)	1432 (61.3)	461 (78.9)	395 (67.6)		325 (55.7)	251 (43.1)	< 0.001*
Diabetic nephropathy (n, %)	480 (20.6)	33 (5.7)	78 (13.4)		143 (24.5)	226 (38.8)	< 0.001*
Renal vascular diseases (n, %)	179 (7.7)	23 (3.9)	43 (7.4)		51 (8.7)	62 (10.6)	< 0.001*
Others (n, %)	244 (10.4)	67 (11.5)	68 (11.6)		65 (11.1)	44 (7.5)	0.069
BMI (kg/m^2^)	21.8 ± 3.2	18.4 ± 1.5	20.5 ± 1.3		22.5 ± 1.4	25.6 ± 2.6	< 0.001*
Systolic pressure (mmHg)	136 ± 20	135 ± 19	137 ± 20		136 ± 20	136 ± 21	0.617
Diastolic pressure (mmHg)	85 ± 14	87 ± 13	86 ± 14		84 ± 14	83 ± 13	< 0.001*
Urine output (ml)	1000 (600–1500)	900 (500–1400)	1000 (600–1500)		1000 (700–1600)	1100 (600–1700)	< 0.001*
**Comorbidities**							
Diabetes (n, %)	564 (24.2)	41 (7.0)	96 (16.4)		170 (29.1)	257 (44.1)	< 0.001*
CVD history (n, %)	782 (33.5)	136 (23.3)	181 (31.0)		213 (36.5)	252 (43.2)	< 0.001*
Hypertension (n, %)	664 (28.4)	79 (13.5)	155 (26.5)		188 (32.2)	242 (41.5)	< 0.001*
**Laboratory parameters**							
Hemoglobin (g/l)	101 ± 22	101 ± 24	101 ± 22		101 ± 22	101 ± 20	0.900
Albumin (g/l)	36.1 ± 5.1	36.9 ± 5.3	36.7 ± 4.9		36.5 ± 5.1	36.6 ± 5.1	0.593
Triglyceride (mg/dl)	124 (89–178)	91 (68–122)		116 (89–156)	130 (98–172)	186 (134–274)	< 0.001*
FBG (mg/dl)	84.6 (75.6–100.8)	79.2 (70.2–86.4)		84.6 (75.6–95.4)	88.2 (77.4–104.4)	95.4 (82.8–138.6)	< 0.001*
TyG index	8.62 (8.23–9.08)	8.17 (7.88–8.49)	8.54 (8.23–8.85)		8.69 (8.38–9.04)	9.20 (8.78–9.65)	< 0.001*
Total cholesterol (mg/dl)	193 ± 52	185 ± 49	192 ± 50		196 ± 50	202 ± 55	< 0.001*
HDL-C (mg/dl)	47.5 ± 16.3	54.4 ± 18.2	49.2 ± 16.5		45.7 ± 13.5	40.8 ± 13.2	< 0.001*
LDL-C (mg/dl)	114 ± 39	108 ± 35	115 ± 37		119 ± 39	116 ± 42	< 0.001*
Serum creatinine (mg/dl)	8.74 ± 3.10	8.82 ± 3.14	8.84 ± 3.20		8.64 ± 2.83	8.68 ± 3.23	0.661
Uric acid (mg/dl)	7.15 ± 1.63	7.03 ± 1.60	7.17 ± 1.64		7.07 ± 1.62	7.32 ± 1.66	0.009*
Urea nitrogen (mg/dl)	49.8 ± 29.2	50.7 ± 21.0	49.2 ± 20.4		50.6 ± 20.2	48.9 ± 19.2	0.240
eGFR (ml/min/1.73 m^2^)	6.73 ± 2.84	6.75 ± 2.71	6.73 ± 2.87		6.70 ± 2.65	6.73 ± 3.12	0.926
hs-CRP (mg/l)	1.69 (0.66–5.31)	0.86 (0.36–2.78)	1.25 (0.24–3.95)		1.58 (0.57–4.84)	2.92 (1.03–7.43)	< 0.001*
**Medicine**							
Antihypertension agents, n (%)	2010 (86.1)	483 (82.7)	498 (85.3)		509 (87.2)	520 (89.2)	0.041*
Antidiabetics agents, n (%)	331 (14.2)	23 (3.9)	48 (8.2)		96 (16.4)	164 (28.1)	< 0.001*
Lipid-lowering agents, n (%)	251 (10.7)	29 (5.0)	46 (7.9)		70 (12.0)	106 (18.2)	< 0.001*

BMI body mass index, CVD cardiovascular disease, FBG fasting blood glucose, TyG triglyceride glucose, HDL-C high density lipoprotein cholesterol, LDL-C low density lipoprotein cholesterol, eGFR estimated glomerular filtration rate, hs-CRP high-sensitivity C-reactive protein

### Factors associated with higher TyG-BMI

Multivariate linear regression analysis showed that advanced age, male sex, history of CVD, history of diabetes, higher hemoglobin level, higher albumin level, higher LDL-C level, higher urine output, and use of lipid-lowering agents were independently associated with higher TyG-BMI after adjusting for age, sex, history of CVD, history of diabetes, urine output, SBP, hemoglobin, albumin, LDL-C, and use of lipid-lowering agents (*P* < 0.05) (Table 2).

**Table 2:** Association between TyG-BMI and reference parameters

Variables	Unstandardized regression coefficient		Standardized regression coefficient	*T*	*P* value
	*B*	Standard error			
Age (years)	0.283	0.056	0.122	5.063	< 0.001*
Sex (male *vs.* female)	–4.114	1.462	–0.059	–2.815	0.005*
History of CVD (yes *vs.* no)	4.595	1.573	0.064	2.921	0.005*
History of diabetes (yes *vs*. no)	21.058	1.906	0.263	11.049	< 0.001*
Systolic pressure (mmHg)	–0.036	0.035	–0.021	–1.035	0.301
Hemoglobin (g/l)	–0.103	0.035	–0.065	–2.929	0.003*
Albumin (g/l)	0.831	0.154	0.124	5.386	< 0.001*
LDL-C (mg/dl)	3.119	0.708	0.092	4.403	< 0.001*
Urine output (ml)	0.008	0.001	0.141	6.806	< 0.001*
Lipid-lowering agents (yes *vs.* no)	10.304	2.169	0.099	4.750	< 0.001*

CVD cardiovascular disease, LDL-C low density lipoprotein cholesterol

### Association of TyG-BMI with CVD and all-cause death

During the follow-up period of 46.6 (22.4–78.0) months, 615 (26.3%) deaths occurred. Furthermore, 426 (18.2%) patients were permanently transferred to hemodialysis, 595 (25.5%) received a kidney transplant, 86 (3.7%) were transferred to other centers, and 60 (2.6%) lost contact with our center. CVD (297; 48.3%) was the dominant cause of death. The remaining causes of death were infection (121; 19.7%), cachexia (36; 5.8%), malignancy (20; 3.3%), other reasons (72; 11.7%), and unknown reasons (69; 11.2%).

**Fig. 2:**
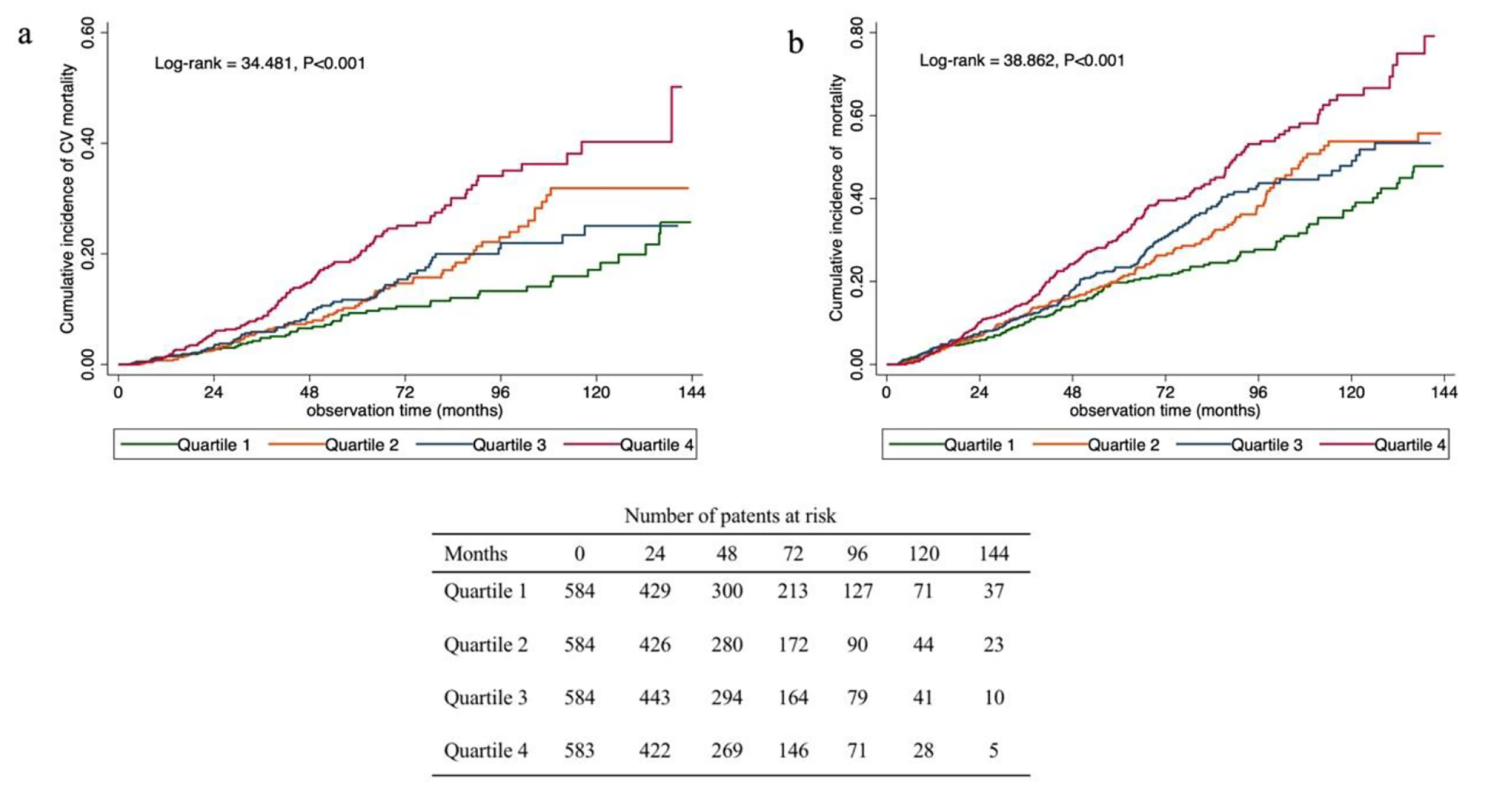
Cumulative incidence function curves for the cumulative incidence of CVD mortality **(a)** and all-cause mortality **(b)** in patients categorized by TyG-BMI quartiles

Kaplan–Meier estimates of CVD and all-cause mortality for patients among the quartiles of TyG-BMI are shown in Fig. 2. At the end of 1, 3, and 5 years, CVD mortality rates were 2.5, 5.1, and 9.7% in the Q1 group; 0.9, 6.1, and 11.0% in the Q2 group; 1.7, 6.2, and 12.1% in the Q3 group; and 1.7, 8.6, and 19.7% in the Q4 group, respectively. Patients with the highest TyG-BMI (Q4 group) had a significantly increased CVD mortality rate compared to those in the Q1 group (*P* < 0.001) (Fig. 2a). The all-cause mortality rates were 4.0, 10.4, and 20.1% in the Q1 group; 2.7, 12.4, and 21.0% in the Q2 group; 4.1, 11.9, and 23.8% in the Q3 group; and 2.9, 14.8, and 31.2% in the Q4 group, respectively. Patients with the highest TyG-BMI (Q4 group) had a higher rate of all-cause mortality than those in the Q1 group (*P* < 0.001) (Fig. 2b).

The results of the Cox regression analysis showed that TyG-BMI in Q4 was markedly associated with an increased risk of CVD mortality (HR 1.51, 95% CI 1.05–2.17; *P* = 0.027) and all-cause mortality (HR 1.36, 95% CI 1.05–1.75; *P* = 0.018) in comparison with Q1 in the final adjusted model. After full adjustment, a TyG-BMI increase of 1 standard deviation (SD) (34.2) was associated with a 28% higher risk (95% CI 1.13–1.45; *P* < 0.001) and a 19% higher risk (95% CI 1.09–1.31; *P* < 0.001) of CVD and all-cause death, respectively (Table 3).

**Table 3:** Association between TyG-BMI and CVD/all-cause mortality

	Model 1		Model 2		Model 3	
	HR (95% CI)	*P* value	HR (95% CI)	*P* value	HR (95% CI)	*P* value
**CVD mortality**						
TyG-BMI (per SD increase )	1.38 (1.24–1.53)	< 0.001*	1.24 (1.10–1.39)	< 0.001*	1.28 (1.13–1.45)	< 0.001*
Quartile 2	1.47 (1.03–2.10)	0.036*	0.97 (0.68–1.40)	0.883	0.93 (0.62–1.37)	0.700
Quartile 3	1.40 (0.98–2.00)	0.068	0.83 (0.58–1.20)	0.323	0.80 (0.54–1.20)	0.280
Quartile 4	2.46 (1.77–3.42)	< 0.001*	1.50 (1.08–2.10)	0.016*	1.51 (1.05–2.17)	0.027*
**All-cause mortality**						
TyG-BMI (per SD increase )	1.28 (1.19–1.38)	< 0.001*	1.21 (1.03–1.22)	0.008*	1.19 (1.09–1.31)	< 0.001*
Quartile 2	1.32 (1.04–1.69)	0.023*	0.92 (0.72–1.18)	0.501	0.91 (0.69–1.19)	0.487
Quartile 3	1.42 (1.12–1.80)	0.004*	0.85 (0.67–1.08)	0.194	0.91 (0.70–1.19)	0.503
Quartile 4	2.00 (1.59–2.51)	< 0.001*	1.23 (0.98–1.55)	0.080	1.36 (1.05–1.75)	0.018*

HR, hazard ratio

CI, confidence interval

SD, standard deviation

Reference group was Quartile 1

Model 1: Unadjusted

Model 2: Adjusted for age, sex, systolic pressure, and history of CVD

Model 3: Adjusted for model 2 covariates and hemoglobin, serum albumin, LDL-C, urine output and use of lipid-lowering agents

*P < 0.05

**Fig. 3:**
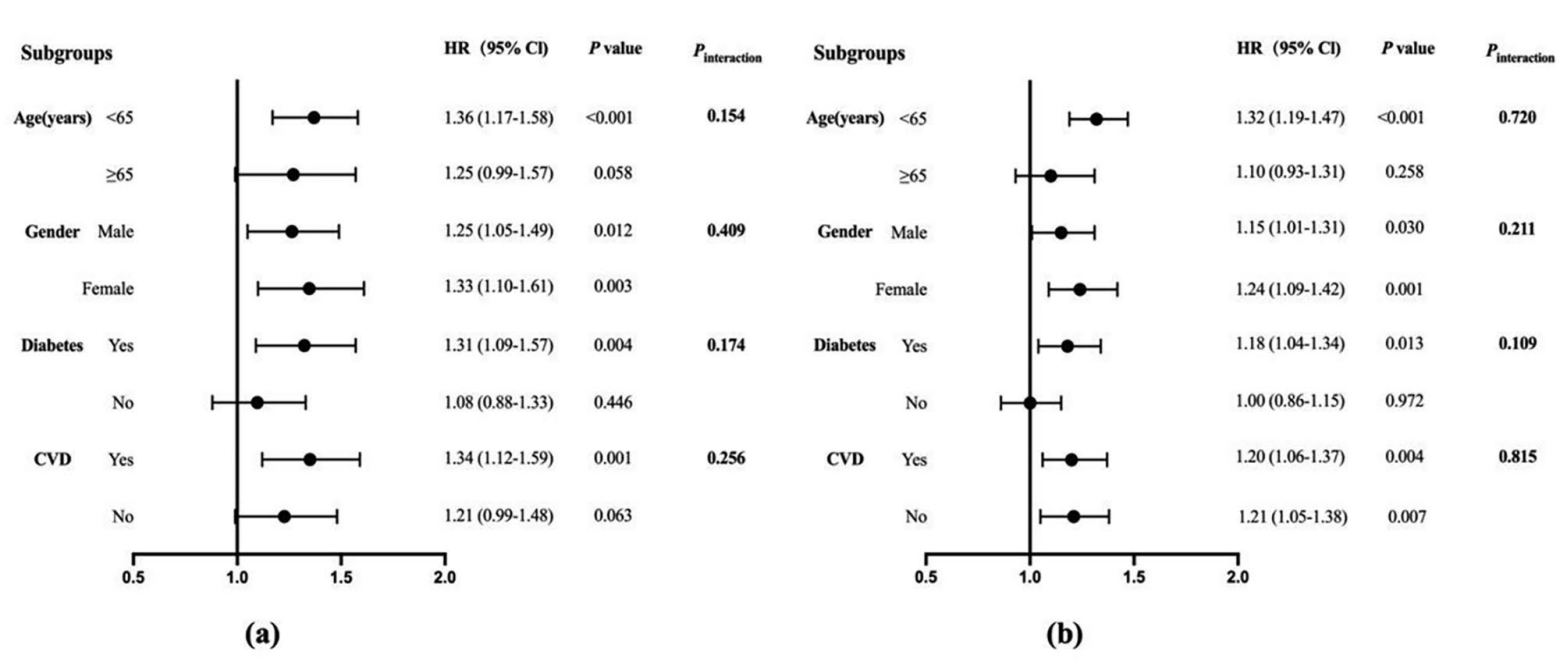
Subgroup analyses. A comparison of the adjusted hazard ratios of CVD mortality **(a)** and all-cause mortality **(b)** for the subgroups is presented by forest plot. Adjusted for age, sex, history of CVD, hemoglobin, albumin, LDL-C, urine output, and lipid-lowering agents for each subgroup (excluding for its own group)

Subgroup analysis showed that higher TyG-BMI was associated with a higher CVD mortality risk in patients aged < 65 years (adjusted HR 1.36, 95% CI 1.17–1.58; *P* < 0.001), those with diabetes (adjusted HR 1.31, 95% CI 1.09–1.57; *P* = 0.004), and those with a history of CVD (adjusted HR 1.34, 95% CI 1.21–1.59; *P* = 0.001) (Fig. 3a). TyG-BMI was also associated analogously with all-cause death risk in patients aged < 65 years (adjusted HR 1.32, 95% CI 1.19–1.47; *P* < 0.001) as well as in those with diabetes (adjusted HR 1.18, 95% CI 1.04–1.34; *P* = 0.013) (Fig. 3b).

## Discussion

In this study, we identified the factors associated with a higher TyG-BMI included advanced age, male sex, history of CVD or diabetes, higher hemoglobin, albumin and LDL-C levels, higher urine output and use of lipid-lowering agents. It was demonstrated that a higher TyG-BMI was significantly associated with elevated odds of CVD and all-cause mortality in patients undergoing PD, especially in those aged < 65 years and those with diabetes even after adjusting for multiple confounders.

Although the homeostasis model assessment of IR (HOMA-IR) is a classic hallmark of IR, its costliness and complexity have prevented its wide usage [16]. Therefore, TyG-BMI was suggested as an effective and convenient substitute marker of IR [7]. Subsequently, the application of TyG-BMI was demonstrated in IR-related diseases, such as prediabetes, diabetes, hypertension, and nonalcoholic fatty liver disease [13, 17–19]. Zhi et al. elucidated a robust link between ischemic stroke and TyG-BMI and found that risk stratification of ischemic stroke patients was improved using this index [12]. Yu et al. reported that TyG-BMI is highly relevant to the severity of coronary artery disease [11]. However, another study involving 3,281 participants found that TyG-BMI was not useful in predicting incident hypertension [20]. In the present study, PD patients with a higher TyG-BMI had an increased rate of CVD death, and TyG-BMI was an independent risk factor associated with CVD mortality in PD patients even after adjusting for clinical parameters. Furthermore, the association was greatest among patients aged < 65 years and those with diabetes. One potential explanation for this phenomenon is that older people tend to suffer from multiple diseases; thus, a single indicator fails to completely estimate their impact. TyG-BMI is derived from lipid and glucose levels and obesity, which are associated with diabetes. Notably, these findings emphasize the applicability of this index in the early identification of CVD risk in patients undergoing PD.

Conventional glucose-containing PD fluids are the principal treatment of choice for most patients undergoing initial dialysis. However, exposure to glucose-containing PD fluids over a long period of time exerts adverse effects on death rates, especially via CVD. Our previous study found that a higher peritoneal dialysate glucose concentration (PDGC) generated detrimental effects on the risk of CVD and all-cause death compared with a low PDGC in patients receiving PD [21]. Glucose disorders and IR are often observed in CKD patients, and the utilization of glucose dialysate exacerbates metabolic abnormalities in PD patients, which drives micro- and macrovascular lesions [22]. Recent clinical research has emphasized the relationship between IR and the progression of CVD in ESRD patients; however, previous reports on the association of CVD mortality with IR among patients receiving PD have inconsistent results [23–25]. ‘Uremic dyslipidemia’ was featured with high serum TC, low HDL-C, and normal LDL-C levels, which are specifically presented in patients with CKD [26]. J.A. et al. showed that high levels of TGs were relevant to adverse CVD outcomes [27]. High TG levels may accelerate oxidative stress, inflammation, and endothelial dysfunction, leading to CKD-related CVD progression [26]. In addition, FBG (> 5.7 mmol/L) was an independent risk factor associated with CVD-free survival in patients who received continuous ambulatory PD [28]. Moreover, adiposity has been verified to be related to cardiometabolic risk factors in the CKD population including in children with CKD [29, 30]. Higher BMI was demonstrated to be closely associated with an increased risk of CVD mortality in the continuous ambulatory PD group [31]. TG, FBG, and BMI affect CVD progression in PD patients [32–34]. However, the association between TG levels, BMI, and CVD mortality remains controversial [35, 36]. Our study indicates that TyG-BMI, which combines these factors, is a clinically valuable surrogate marker of CVD mortality in patients undergoing PD. In Chinese PD patients, a high TG concentration was related to poor patient survival [37]. Another study implemented on the international Monitoring Dialysis Outcomes database showed inverse results [38]. Our recent study emphasized that higher baseline FBG levels (≥ 7mmol/L) were associated with all-cause mortality in PD patients with higher LDL-C levels [39]. In addition, by enrolling 274 Asian PD patients, Kiran et al. elucidated that BMI had a U-shaped link to mortality [40]. The above findings suggest that the TyG-BMI components (triglyceride, fasting glucose, and BMI) were all linked to patients’ clinical outcome. In the present study, TyG-BMI was independently associated with all-cause mortality. To the best of our knowledge, no studies to date have investigated the correlation between TyG-BMI and CVD risk and all-cause death in patients undergoing PD.

### Study strengths and limitations

In this study, we found first an independent relationship between TyG-BMI and CVD mortality and all-cause mortality in a large PD population. Nevertheless, this study has some limitations. First, some confounding factors, such as glucose prescription, which may influence patient survival, were not included in the analysis as the data are difficult to obtain; the results may therefore be biased to some extent. Second, only baseline TyG-BMI data were analyzed; therefore, longitudinal cohort studies are warranted to investigate whether the association between TyG-BMI and mortality persists over time. Last, owing to the lack of data on insulin levels during follow-up, we did not examine HOMA-IR or compare it with TyG-BMI.

## Conclusions

A cohort study of Chinese patients with sustained PD concluded that an elevated TyG-BMI was significantly related to an increased risk of CVD and all-cause mortality. TyG-BMI might be a valuable tool for identifying PD patients at high risk of CVD mortality. Thus, TyG-BMI could be recommended as part of routine surveillance during the follow-up of PD patients aged < 65 years and those with diabetes mellitus.

### Electronic supplementary material

Below is the link to the electronic supplementary material.

Supplementary Material 1

## Data Availability

The datasets used and/or analyzed during the current study are available from the corresponding author on reasonable request.

## Abbreviations

TyG-BMI: triglyceride glucose-body mass index
CVD: cardiovascular disease
PD: peritoneal dialysis
BMI: body mass index
HR: hazard ratio
CI: confidence interval
SD: standard deviation
CKD: chronic kidney disease
TG: triglyceride
FBG: fasting blood glucose
IR: insulin resistance
ESRD: end-stage renal disease
TC: total cholesterol
TG: triglyceride
LDL-C: low-density lipoprotein cholesterol
HDL-C: high-density lipoprotein cholesterol
hs-CRP: high-sensitivity C-reactive protein
eGFR: estimated glomerular filtration rate
SBP: systolic blood pressure
DBP: diastolic blood pressure
Q: quartile
HOMA-IR: homeostasis model assessment of insulin resistance
PDGC: peritoneal dialysate glucose concentration
