# Supplementary material for: Triglyceride glucose-body mass index and cardiovascular mortality in patients undergoing peritoneal dialysis: a retrospective cohort study
Source: Lipids Health Dis. 2023 Sep 5;22:143. doi: 10.1186/s12944-023-01892-2 (PMC10478298; doi:10.1186/s12944-023-01892-2)
Supplement: Supplementary file 1 — Supplementary Material 1 [file 12944_2023_1892_MOESM1_ESM.docx]

**Triglyceride glucose-body mass index and cardiovascular mortality in patients undergoing peritoneal dialysis: A retrospective cohort study**

Cuixia Zhan^1,2^^†^, Yuan Peng^1,2,3†^, Hongjian Ye^1,2^, Xiangwen Diao^1,2^, Chunyan Yi^1,2^, Qunying Guo^1,2^, Wei Chen^1,2^, Xiao Yang^1,2^*

^1^Department of Nephrology, The First Affiliated Hospital, Sun Yat-sen University, Guangzhou, 510080, China

^2^NHC Key Laboratory of Clinical Nephrology (Sun Yat-Sen University) and Guangdong Provincial Key Laboratory of Nephrology, Guangzhou, 510080, China

^3^Department of Nephrology, Ganzhou People's Hospital (The Affiliated Ganzhou Hospital of Nanchang University), Ganzhou, 341000, China.

^†^Cuixia Zhan and Yuan Peng contributed equally to this study.

Short Title: Triglyceride glucose-body mass index and cardiovascular mortality in PD patients

*Corresponding Author: Xiao Yang, M.D. & Ph.D.

Full name: Xiao Yang

Department: Nephrology

Institute/University/Hospital: The First Affiliated Hospital, Sun Yat-sen University

Street Name & Number: 58th, Zhongshan Road II

City, State, Postal code, Country: Guangzhou, 510080, China

Tel: +86-20-87755766-8843

E-mail: yxiao@mail.sysu.edu.cn

**Abstract**

**Background:** Recent studies have shown that triglyceride glucose-body mass index (TyG-BMI) is associated with the risk of ischemic stroke and coronary artery disease. However, little attention has been given to the association between TyG-BMI and cardiovascular disease (CVD) mortality in patients undergoing peritoneal dialysis (PD). Therefore, this study aimed to explore the relationship between TyG-BMI and CVD mortality in southern Chinese patients undergoing PD.

**Methods:** Incident patients receiving PD from January 1, 2006, to December 31, 2018, with baseline serum triglyceride, glucose, and body mass index (BMI) information, were recruited for this single-center retrospective cohort study. TyG-BMI was calculated based on fasting plasma glucose, triglyceride, and BMI values. The association between TyG-BMI, CVD and all-cause mortality was evaluated using a multivariate-adjusted Cox proportional hazard regression model.

**Results:** Of 2,335 patients, the mean age was 46.1±14.8 years; 1,382 (59.2%) were male, and 564 (24.2%) had diabetes. The median TyG-BMI was 183.7 (165.5–209.2). Multivariate linear regression showed that advanced age, male sex, history of CVD, higher levels of albumin and low-density lipoprotein cholesterol, and higher urine output were correlated with a higher TyG-BMI (*P*<0.05). During a median follow-up period of 46.6 (22.4–78.0) months, 615 patients died, of whom 297 (48.2%) died as a result of CVD. After adjusting for demographics and comorbidities, TyG-BMI was significantly associated with an increased risk of CVD mortality (hazard ratio [HR] 1.51, 95% confidence interval [CI] 1.05–2.17) and all-cause mortality (HR 1.36, 95% CI 1.05–1.75). After full adjustment, the 28% risk of CVD mortality (HR 1.28, 95% CI 1.13‒1.45) and 19% risk of all-cause mortality were elevated (HR 1.19, 95% CI 1.09‒1.31) when TyG-BMI increased by 1 stand deviation (SD) (34.2).

**Conclusions:** A higher baseline TyG-BMI was independently associated with an increased risk of CVD and all-cause mortality in patients receiving PD.

**Keywords:** TyG-BMI; cardiovascular mortality; peritoneal dialysis.

**Background**

Cardiovascular disease (CVD) accounts for 52.7% of deaths in the peritoneal dialysis (PD) population [1]. In addition to classic factors (dyslipidemia, diabetes, and obesity) and chronic kidney disease (CKD)-related factors (inflammation, malnutrition, and fluid overload), PD-specific factors (peritoneal glucose exposure, advanced glycation end products, and bioincompatible solutions) play pivotal roles in the increased risk of CVD in dialysis patients [2, 3]. Despite substantial advances in the treatment and management of CVD in the PD population in recent years, mortality remains high, suggesting that there are huge obstacles that need to be overcome to prevent or delay the development of CVD [4-6].

A composite measure of plasma triglyceride (TG), fasting blood glucose (FBG), and body mass index (BMI), TyG-BMI, has recently been identified as a substitute target for insulin resistance (IR) [7-9], which has been identified as a risk factor associated with CVD [10]. The index simultaneously combines glucose, lipid levels and BMI, reflecting multiple critical factors in the management of PD. Recent studies have shown that TyG-BMI was associated with the risk of ischemic stroke, hypertension, and coronary artery disease [11-13]. However, its relationship with CVD mortality in the PD population remains unclear. Therefore, this study aimed to explore the association of TyG-BMI with CVD mortality based on a large-cohort Chinese PD population.

**Methods**

**Study design and participants**

This retrospective cohort study was conducted at a large PD center in southern China. From January 1, 2006, to December 31, 2018, end-stage renal disease (ESRD) patients who were catheterized and followed up at our PD center were recruited. Patients under the age of 18 years at the onset of PD treatment and those who had malignant tumors, suspended PD treatment within 3 months, had a failed kidney transplantation or were transferred from hemodialysis were excluded from the study. The project was approved by the Human Ethics Committee of the First Affiliated Hospital of Sun Yat-sen University.

**Data collection**

Information on demographics and relevant disease conditions during patient admission to the PD center was collected. Demographic characteristics included age, sex, primary cause of ESRD, and comorbidities (diabetes mellitus, history of CVD, and hypertension). The participants were considered to have diabetes mellitus if they were diagnosed with diabetes by an endocrinologist or were taking antidiabetic medication. Participants with any of the following events were considered to have CVD: coronary artery bypass grafting, myocardial infarction, heart failure, angioplasty, stroke, or angina pectoris [14]. Blood pressure levels that exceeded 140/90 mmHg after repeated measurements in a rest state or hypotensive drug use were defined as hypertension.

Laboratory parameters were acquired during the initial three months of PD treatment. Fasting blood samples were collected and analyzed in the hospital laboratory. Levels of hemoglobin, serum albumin, FBG, total cholesterol (TC), TG, low-density lipoprotein cholesterol (LDL-C), high-density lipoprotein cholesterol (HDL-C), serum urea nitrogen, serum creatinine, uric acid, and high-sensitivity C-reactive protein (hs-CRP) were assessed. The estimated glomerular filtration rate (eGFR) was calculated with the CKD epidemiology collaboration formula. BMI, systolic blood pressure (SBP), diastolic blood pressure (DBP), and urine output were measured synchronously. Medication history, including the use of antihypertensive agents, hypoglycemic agents, and lipid-lowering medications, was recorded.

The equation for calculating the index is as follows:

TyG=Ln[(1/2FBG(mg/dL))×TG(mg/dL)] [15],

BMI=weight divided by height^2^,

TyG-BMI=TyG ×BMI [7].

**Study outcomes and definition**

The primary and secondary end points were CVD and all-cause mortality, respectively. The criteria for CVD death were death attributed to cerebrovascular disorders, anoxic encephalopathy, peripheral arterial disease, ischemic brain injury, congestive heart failure, cardiomyopathy, cardiac arrhythmia, cardiac arrest, acute myocardial infarction, or atherosclerotic heart disease [14]. The cause of death was identified by the comprehensive management team of the PD center, which consisted of primary and senior professors.

All patients were followed up until death, conversion to hemodialysis therapy, receipt of a kidney transplant, transfer to another center, loss to follow-up, or December 31, 2021.

**Statistical analysis**

According to quartiles (Q) of TyG-BMI levels, participants were divided into four groups: Q1, <165.5; Q2, 165.5-<183.7; Q3, 183.7-<209.2; and Q4, >209.2. The results are reported as frequencies and percentages for categorical data, means and standard deviations for normally distributed data, and medians and interquartile ranges for nonnormally distributed data. Differences between groups were compared by Student’s t test, Mann–Whitney U test or *χ^2^* test.

A linear regression model was used to analyze the relevant parameters of TyG-BMI. Kaplan–Meier curves were plotted to analyze survival time, and the distributions of survival among TyG-BMI quartiles were assessed by a log-rank test. The association between TyG-BMI and CVD and all-cause mortality was examined in Cox proportional hazards models. TG, FBG, and BMI comprise the TyG-BMI, which is closely related to diabetes. Therefore, the adjusted model did not include diabetes to avoid apparent bias caused by the correcting variable. The results are shown as hazard ratios (HRs) and 95% confidence intervals (CIs). To further explore the association between TyG-BMI and CVD and all-cause death, subgroup analyses were performed using clinical parameters, and the results are shown in a forest plot. All statistical analyses were performed using SPSS software version 26.0 (IBM Corp., Armonk, NY, USA); a value of *P*<0.05 was considered as statistically significant.

**Results**

**Baseline characteristics of participants**

In total, 2,689 patients undergoing PD who were catheterized at our PD center were recruited. Of these, 354 patients who were aged <18 years, transferred from maintenance hemodialysis, underwent failed kidney transplantation, had malignant tumors, received PD for less than 3 months, or lacked baseline TyG-BMI data were excluded. The remaining 2,335 participants were eligible for the final analysis (Figure 1).

Participants’ mean age was 46.1±14.8 years; 1,382 (59.2%) were male, and diabetic patients accounted for 24.2% (n=564). The primary renal diseases were chronic glomerulonephritis (61.3%), diabetic nephropathy (20.6%) and hypertension (7.7%). Baseline TyG-BMI ranged from 82.0 to 380.0 (median 183.7, interquartile range 165.5–209.2). The baseline characteristics of the participants by quartiles of TyG-BMI are presented in Table 1. Compared with patients in Q1, patients with higher TyG-BMI levels were older; had a higher incidence of diabetes, CVD, and hypertension; had a higher proportion of males; and had increased urine output and higher TC, LDL-C, uric acid, and hs-CRP levels but lower DBP and HDL-C levels (P<0.05) (Table 1). There was no obvious distinction among the groups regarding SBP, hemoglobin, albumin, serum creatinine, urea nitrogen, and eGFR levels (P>0.05) (Table 1).

**Factors associated with higher** **TyG-BMI**

Multivariate linear regression analysis showed that older age, male sex, history of CVD, history of diabetes, higher hemoglobin level, higher albumin level, higher LDL-C level, and higher urine output were independently associated with higher TyG-BMI after adjusting for age, sex, history of CVD, history of diabetes, urine output, SBP, hemoglobin, albumin, LDL-C, and use of lipid-lowering agents (*P*<0.05) (Table 2).

**Association of TyG-BMI with CVD and all-cause death**

During the follow-up period of 46.6 (22.4–78.0) months, 615 (26.3%) deaths occurred. Furthermore, 426 (18.2%) patients were permanently transferred to hemodialysis, 595 (25.5%) received a kidney transplant, 86 (3.7%) were transferred to other centers, and 60 (2.6%) lost contact with our center. CVD (297; 48.3%) was the dominant cause of death. The remaining causes of death were infection (121; 19.7%), cachexia (36; 5.8%), malignancy (20; 3.3%), other reasons (72; 11.7%), and unknown reasons (69; 11.2%).

Kaplan‒Meier estimates of CVD and all-cause mortality for patients among the quartiles of TyG-BMI are shown in Figure 2. At the end of 1, 3, and 5 years, CVD mortality rates were 2.5, 5.1, and 9.7% in the Q1 group; 0.9, 6.1, and 11.0% in the Q2 group; 1.7, 6.2, and 12.1% in the Q3 group; and 1.7, 8.6, and 19.7% in the Q4 group, respectively. Patients with the highest TyG-BMI (Q4 group) had a significantly increased CVD mortality rate compared to those in the Q1 group (*P*<0.001) (Figure 2a). The all-cause mortality rates were 4.0, 10.4, and 20.1% in the Q1 group; 2.7, 12.4, and 21.0% in the Q2 group; 4.1, 11.9, and 23.8% in the Q3 group; and 2.9, 14.8, and 31.2% in the Q4 group, respectively. Patients with the highest TyG-BMI (Q4 group) had a higher rate of all-cause mortality than those in the Q1 group (*P*<0.001) (Figure 2b).

The results of the Cox regression analysis showed that TyG-BMI in Q4 was markedly associated with an increased risk of CVD mortality (HR 1.51, 95% CI 1.05–2.17; *P*=0.027) and all-cause mortality (HR 1.36, 95% CI 1.05–1.75; *P*=0.018) in comparison with Q1 in the final adjusted model. After full adjustment, a TyG-BMI increase of 1 standard deviation (SD) (34.2) was associated with a 28% higher risk (95% CI=1.13–1.45; *P*<0.001) and a 19% higher risk (95% CI=1.09–1.31; *P*<0.001) of CVD and all-cause death, respectively (Table 3).

Subgroup analysis showed that higher TyG-BMI was associated with a higher CVD mortality risk in patients aged <65 years (adjusted HR 1.36, 95% CI 1.17–1.58; *P*<0.001), those with diabetes (adjusted HR 1.31, 95% CI 1.09–1.57; *P*=0.004), and those with a history of CVD (adjusted HR 1.34, 95% CI 1.21–1.59; *P*=0.001) (Figure 3a). TyG-BMI was also associated analogously with all-cause death risk in patients aged <65 years (adjusted HR 1.32, 95% CI 1.19–1.47; *P*<0.001) as well as in those with diabetes (adjusted HR 1.18, 95% CI 1.04–1.34; *P*=0.013) (Figure 3b).

**Discussion**

In this study, we identified the factors associated with a higher TyG-BMI included advanced age, male sex, history of CVD or diabetes, higher hemoglobin, albumin and LDL-C levels, and higher urine output. It was demonstrated that a higher TyG-BMI was significantly associated with elevated odds of CVD and all-cause mortality in patients undergoing PD, especially in those aged <65 years and those with diabetes even after adjusting for multiple confounders.

Although the homeostasis model assessment of IR (HOMA-IR) is a classic hallmark of IR, its costliness and complexity have prevented its wide usage [16]. Therefore, TyG-BMI was suggested as an effective and convenient substitute marker of IR [7]. Subsequently, the application of TyG-BMI was demonstrated in IR-related diseases, such as prediabetes, diabetes, hypertension, and nonalcoholic fatty liver disease [13, 17-19]. Zhi et al. elucidated a robust link between ischemic stroke and TyG-BMI and found that risk stratification of ischemic stroke patients was improved using this index [12]. Yu et al. reported that TyG-BMI is highly relevant to the severity of coronary artery disease [11]. However, another study involving 3,281 participants found that TyG-BMI was not useful in predicting incident hypertension [20]. In the present study, PD patients with a higher TyG-BMI had an increased rate of CVD death, and TyG-BMI was an independent risk factor associated with CVD mortality in PD patients even after adjusting for clinical parameters. Furthermore, the association was greatest among patients aged <65 years and those with diabetes. One potential explanation for this phenomenon is that older people tend to suffer from multiple diseases; thus, a single indicator fails to completely estimate their impact. TyG-BMI is derived from lipid and glucose levels and obesity, which are associated with diabetes. Notably, these findings emphasize the applicability of this index in the early identification of CVD risk in patients undergoing PD.

Conventional glucose-containing PD fluids are the principal treatment of choice for most patients undergoing initial dialysis. However, exposure to glucose-containing PD fluids over a long period of time exerts adverse effects on death rates, especially via CVD. Our previous study found that a higher peritoneal dialysate glucose concentration (PDGC) generated detrimental effects on the risk of CVD and all-cause death compared with a low PDGC in patients receiving PD [21]. Glucose disorders and IR are often observed in CKD patients, and the utilization of glucose dialysate exacerbates metabolic abnormalities in PD patients, which drives micro- and macrovascular lesions [22]. Recent clinical research has emphasized the relationship between IR and the progression of CVD in ESRD patients; however, previous reports on the association of CVD mortality with IR among patients receiving PD have inconsistent results [23-25]. ‘Uremic dyslipidemia’ was featured with high serum TC, low HDL-C, and normal LDL-C levels, which are specifically presented in patients with CKD [26]. J.A. et al. showed that high levels of TGs were relevant to adverse CVD outcomes [27]. High TG levels may accelerate oxidative stress, inflammation, and endothelial dysfunction, leading to CKD-related CVD progression [26]. In addition, FBG (>5.7 mmol/L) was an independent risk factor associated with CVD-free survival in patients who received continuous ambulatory PD [28]. Moreover, adiposity has been verified to be related to cardiometabolic risk factors in the CKD population including in children with CKD [29, 30]. Higher BMI was demonstrated to be closely associated with an increased risk of CVD mortality in the continuous ambulatory PD group [31]. TG, FBG, and BMI affect CVD progression in PD patients [32-34]. However, the association between TG levels, BMI, and CVD mortality remains controversial [35, 36]. Our study indicates that TyG-BMI, which combines these factors, is a clinically valuable surrogate marker of CVD mortality in patients undergoing PD. In Chinese PD patients, a high TG concentration was related to poor patient survival [37]. Another study implemented on the international Monitoring Dialysis Outcomes database showed inverse results [38]. Our recent study emphasized that higher baseline FBG levels (≥7mmol/L) were associated with all-cause mortality in PD patients with higher LDL-C levels [39]. In addition, by enrolling 274 Asian PD patients, Kiran et al. elucidated that BMI had a U-shaped link to mortality [40]. The above findings suggest that the TyG-BMI components (triglyceride, fasting glucose, and BMI) were all linked to patients’ clinical outcome. In the present study, TyG-BMI was independently associated with all-cause mortality. To the best of our knowledge, no studies to date have investigated the correlation between TyG-BMI and CVD risk and all-cause death in patients undergoing PD.

**Study strengths and limitations**

In this study, we found first an independent relationship between TyG-BMI and CVD mortality and all-cause mortality in a large PD population. Nevertheless, this study has some limitations. First, some confounding factors, such as glucose prescription, which may influence patient survival, were not included in the analysis as the data are difficult to obtain; the results may therefore be biased to some extent. Second, only baseline TyG-BMI data were analyzed; therefore, longitudinal cohort studies are warranted to investigate whether the association between TyG-BMI and mortality persists over time. Last, owing to the lack of data on insulin levels during follow-up, we did not examine HOMA-IR or compare it with TyG-BMI.

**Conclusions**

A cohort study of Chinese patients with sustained PD concluded that an elevated TyG-BMI was significantly related to an increased risk of CVD and all-cause mortality. TyG-BMI might be a valuable tool for identifying PD patients at high risk of CVD mortality. Thus, TyG-BMI could be recommended as part of routine surveillance during the follow-up of PD patients aged <65 years and those with diabetes mellitus.

**List of abbreviations**

TyG-BMI, triglyceride glucose-body mass index; CVD, cardiovascular disease; PD, peritoneal dialysis; BMI, body mass index; HR, hazard ratio; CI, confidence interval; SD, standard deviation; CKD, chronic kidney disease; TG, triglyceride; FBG, fasting blood glucose; IR, insulin resistance; ESRD, end-stage renal disease; TC, total cholesterol; TG, triglyceride; LDL-C, low-density lipoprotein cholesterol; HDL-C, high-density lipoprotein cholesterol; hs-CRP, high-sensitivity C-reactive protein; eGFR, estimated glomerular filtration rate; SBP, systolic blood pressure; DBP, diastolic blood pressure; Q, quartile; HOMA-IR, homeostasis model assessment of insulin resistance; PDGC, peritoneal dialysate glucose concentration.

**Availability of data and materials**

The datasets used and/or analyzed during the current study are available from the corresponding author on reasonable request.

**Declarations**

**Ethics approval and consent to participate**

This study protocol was conducted in compliance with the ethical principles of the Helsinki Declaration and approved by the Human Ethics Committee of Sun Yat-sen University [Ethics Review (2016) NO.215]. Written informed consent was obtained from all the participants.

**Competing interests**

The authors declare no competing interests.

**Consent for publication**

All the authors listed have approved the manuscript for publication.

**Funding**

This work was supported by Investigatro Initiated Research (IIR) Funding of Baxter (IIR no. 22CEC3RCAPAC1002), Guangdong Provincial Key Laboratory of Nephrology (Grant no. 2020B1212060028), and NHC Key Laboratory of Clinical Nephrology (Sun Yat-Sen University).

**Authors’ contributions**

Xiao Yang proposed the concept of the study. Cuixia Zhan analyzed and drafted the article and revised it under the supervision of Professor Xiao Yang. Yuan Peng, Hongjian Ye, Chunyan Yi, Xiangwen Diao, and Qunying Guo collected the data and contributed to the discussion of the results. All authors approved the final version of the manuscript.

**Acknowledgments**We are grateful to the nurses in our PD center for their great contribution in patient management and database maintenance.

**References**

1. Bello AK, Okpechi IG, Osman MA, Cho Y, Cullis B, Htay H, Jha V, Makusidi MA, McCulloch M, Shah N, et al: **Epidemiology of peritoneal dialysis outcomes.** *Nat Rev Nephrol* 2022**:**1-15.

2. Jegatheesan D, Cho Y, Johnson DW: **Clinical Studies of Interventions to Mitigate Cardiovascular Risk in Peritoneal Dialysis Patients.** *Semin Nephrol* 2018, **38:**277-290.

3. Krediet RT, Balafa O: **Cardiovascular risk in the peritoneal dialysis patient.** *Nat Rev Nephrol* 2010, **6:**451-460.

4. Matsushita K, Ballew SH, Wang AY, Kalyesubula R, Schaeffner E, Agarwal R: **Epidemiology and risk of cardiovascular disease in populations with chronic kidney disease.** *Nat Rev Nephrol* 2022, **18:**696-707.

5. Wang AY, Brimble KS, Brunier G, Holt SG, Jha V, Johnson DW, Kang SW, Kooman JP, Lambie M, McIntyre C, et al: **ISPD Cardiovascular and Metabolic Guidelines in Adult Peritoneal Dialysis Patients Part I - Assessment and Management of Various Cardiovascular Risk Factors.** *Perit Dial Int* 2015, **35:**379-387.

6. Wang AY, Brimble KS, Brunier G, Holt SG, Jha V, Johnson DW, Kang SW, Kooman JP, Lambie M, McIntyre C, et al: **ISPD Cardiovascular and Metabolic Guidelines in Adult Peritoneal Dialysis Patients Part II - Management of Various Cardiovascular Complications.** *Perit Dial Int* 2015, **35:**388-396.

7. Er LK, Wu S, Chou HH, Hsu LA, Teng MS, Sun YC, Ko YL: **Triglyceride Glucose-Body Mass Index Is a Simple and Clinically Useful Surrogate Marker for Insulin Resistance in Nondiabetic Individuals.** *PLoS One* 2016, **11:**e0149731.

8. Lim J, Kim J, Koo SH, Kwon GC: **Comparison of triglyceride glucose index, and related parameters to predict insulin resistance in Korean adults: An analysis of the 2007-2010 Korean National Health and Nutrition Examination Survey.** *PLoS One* 2019, **14:**e0212963.

9. Lee J, Kim B, Kim W, Ahn C, Choi HY, Kim JG, Kim J, Shin H, Kang JG, Moon S: **Lipid indices as simple and clinically useful surrogate markers for insulin resistance in the U.S. population.** *Sci Rep* 2021, **11:**2366.

10. Ginsberg HN: **Insulin resistance and cardiovascular disease.** *J Clin Invest* 2000, **106:**453-458.

11. Zhang Y, Wang R, Fu X, Song H: **Non-insulin-based insulin resistance indexes in predicting severity for coronary artery disease.** *Diabetol Metab Syndr* 2022, **14:**191.

12. Du Z, Xing L, Lin M, Sun Y: **Estimate of prevalent ischemic stroke from triglyceride glucose-body mass index in the general population.** *BMC Cardiovasc Disord* 2020, **20:**483.

13. Deng D, Chen C, Wang J, Luo S, Feng Y: **Association between triglyceride glucose-body mass index and hypertension in Chinese adults: A cross-sectional study.** *J Clin Hypertens (Greenwich)* 2023.

14. Wu H, Xiong L, Xu Q, Wu J, Huang R, Guo Q, Mao H, Yu X, Yang X: **Higher serum triglyceride to high-density lipoprotein cholesterol ratio was associated with increased cardiovascular mortality in female patients on peritoneal dialysis.** *Nutr Metab Cardiovasc Dis* 2015, **25:**749-755.

15. Simental-Mendía LE, Rodríguez-Morán M, Guerrero-Romero F: **The product of fasting glucose and triglycerides as surrogate for identifying insulin resistance in apparently healthy subjects.** *Metab Syndr Relat Disord* 2008, **6:**299-304.

16. Tam CS, Xie W, Johnson WD, Cefalu WT, Redman LM, Ravussin E: **Defining insulin resistance from hyperinsulinemic-euglycemic clamps.** *Diabetes Care* 2012, **35:**1605-1610.

17. Song B, Zhao X, Yao T, Lu W, Zhang H, Liu T, Liu C, Wang K: **Triglyceride Glucose-Body Mass Index and Risk of Incident Type 2 Diabetes Mellitus in Japanese People With Normal Glycemic Level: A Population-Based Longitudinal Cohort Study.** *Front Endocrinol (Lausanne)* 2022, **13:**907973.

18. Jiang C, Yang R, Kuang M, Yu M, Zhong M, Zou Y: **Triglyceride glucose-body mass index in identifying high-risk groups of pre-diabetes.** *Lipids Health Dis* 2021, **20:**161.

19. Sheng G, Lu S, Xie Q, Peng N, Kuang M, Zou Y: **The usefulness of obesity and lipid-related indices to predict the presence of Non-alcoholic fatty liver disease.** *Lipids Health Dis* 2021, **20:**134.

20. Yuan Y, Sun W, Kong X: **Comparison between distinct insulin resistance indices in measuring the development of hypertension: The China Health and Nutrition Survey.** *Front Cardiovasc Med* 2022, **9:**912197.

21. Wen Y, Guo Q, Yang X, Wu X, Feng S, Tan J, Xu R, Yu X: **High glucose concentrations in peritoneal dialysate are associated with all-cause and cardiovascular disease mortality in continuous ambulatory peritoneal dialysis patients.** *Perit Dial Int* 2015, **35:**70-77.

22. Fortes PC, de Moraes TP, Mendes JG, Stinghen AE, Ribeiro SC, Pecoits-Filho R: **Insulin resistance and glucose homeostasis in peritoneal dialysis.** *Perit Dial Int* 2009, **29 Suppl 2:**S145-148.

23. Li Y, Zhang L, Gu Y, Hao C, Zhu T: **Insulin resistance as a predictor of cardiovascular disease in patients on peritoneal dialysis.** *Perit Dial Int* 2013, **33:**411-418.

24. Yoon CY, Lee MJ, Kee YK, Lee E, Joo YS, Han IM, Han SG, Oh HJ, Park JT, Han SH, et al: **Insulin resistance is associated with new-onset cardiovascular events in nondiabetic patients undergoing peritoneal dialysis.** *Kidney Res Clin Pract* 2014, **33:**192-198.

25. Sánchez-Villanueva R, Estrada P, del Peso G, Grande C, Díez JJ, Iglesias P, González E, Aguilar-Rodríguez A, Selgas R, Bajo MA: **Repeated analysis of estimated insulin resistance using the HOMAIR index in nondiabetic patients on peritoneal dialysis and its relationship with cardiovascular disease and mortality.** *Nefrologia* 2013, **33:**85-92.

26. Speer T, Ridker PM, von Eckardstein A, Schunk SJ, Fliser D: **Lipoproteins in chronic kidney disease: from bench to bedside.** *Eur Heart J* 2021, **42:**2170-2185.

27. Lamprea-Montealegre JA, Staplin N, Herrington WG, Haynes R, Emberson J, Baigent C, de Boer IH: **Apolipoprotein B, Triglyceride-Rich Lipoproteins, and Risk of Cardiovascular Events in Persons with CKD.** *Clin J Am Soc Nephrol* 2020, **15:**47-60.

28. Xu L, Hu X, Chen W: **Fibroblast growth factor-23 correlates with advanced disease conditions and predicts high risk of major adverse cardiac and cerebral events in end-stage renal disease patients undergoing continuous ambulatory peritoneal dialysis.** *J Nephrol* 2019, **32:**307-314.

29. Navaneethan SD, Kirwan JP, Remer EM, Schneider E, Addeman B, Arrigain S, Horwitz E, Fink JC, Lash JP, McKenzie CA, et al: **Adiposity, Physical Function, and Their Associations With Insulin Resistance, Inflammation, and Adipokines in CKD.** *Am J Kidney Dis* 2021, **77:**44-55.

30. Brady TM, Roem J, Cox C, Schneider MF, Wilson AC, Furth SL, Warady BA, Mitsnefes M: **Adiposity, Sex, and Cardiovascular Disease Risk in Children With CKD: A Longitudinal Study of Youth Enrolled in the Chronic Kidney Disease in Children (CKiD) Study.** *Am J Kidney Dis* 2020, **76:**166-173.

31. Xiong L, Cao S, Xu F, Zhou Q, Fan L, Xu Q, Yu X, Mao H: **Association of Body Mass Index and Body Mass Index Change with Mortality in Incident Peritoneal Dialysis Patients.** *Nutrients* 2015, **7:**8444-8455.

32. Yu D, Cai Y, Chen Y, Chen T, Qin R, Zhao Z, Simmons D: **Development and validation of risk prediction models for cardiovascular mortality in Chinese people initialising peritoneal dialysis: a cohort study.** *Sci Rep* 2018, **8:**1966.

33. Lluesa JH, López-Romero LC, Monzó JJB, Marugán MR, Boyano IV, Rodríguez-Espinosa D, Gómez-Bori A, Orient AS, Such RD, Perez PS, Jaras JH: **Lipidic profiles of patients starting peritoneal dialysis suggest an increased cardiovascular risk beyond classical dyslipidemia biomarkers.** *Sci Rep* 2022, **12:**16394.

34. Li W, Xu R, Wang Y, Shen J, Li Z, Yu X, Mao H: **Association of body mass index and uncontrolled blood pressure with cardiovascular mortality in peritoneal dialysis patients.** *J Hum Hypertens* 2019, **33:**106-114.

35. Soohoo M, Hashemi L, Hsiung JT, Moradi H, Budoff MJ, Kovesdy CP, Kalantar-Zadeh K, Streja E: **Risk of Atherosclerotic Cardiovascular Disease and Nonatherosclerotic Cardiovascular Disease Hospitalizations for Triglycerides Across Chronic Kidney Disease Stages Among 2.9 Million US Veterans.** *J Am Heart Assoc* 2021, **10:**e022988.

36. Kim YK, Kim SH, Kim HW, Kim YO, Jin DC, Song HC, Choi EJ, Kim YL, Kim YS, Kang SW, et al: **The association between body mass index and mortality on peritoneal dialysis: a prospective cohort study.** *Perit Dial Int* 2014, **34:**383-389.

37. Zhang F, Liu H, Gong X, Liu F, Peng Y, Cheng M, Zhang H, Liu Y, Liu Y, Guo C: **Risk factors for mortality in Chinese patients on continuous ambulatory peritoneal dialysis.** *Perit Dial Int* 2015, **35:**199-205.

38. Kaysen GA, Ye X, Raimann JG, Wang Y, Topping A, Usvyat LA, Stuard S, Canaud B, van der Sande FM, Kooman JP, Kotanko P: **Lipid levels are inversely associated with infectious and all-cause mortality: international MONDO study results.** *J Lipid Res* 2018, **59:**1519-1528.

39. Xu Y, Zhong Z, Li Y, Li Z, Zhou Y, Li Z, Mao H: **Interaction effect between fasting plasma glucose and lipid profiles on mortality of peritoneal dialysis patients.** *Clin Kidney J* 2023, **16:**727-734.

40. Kiran VR, Zhu TY, Yip T, Lui SL, Lo WK: **Body mass index and mortality risk in Asian peritoneal dialysis patients in Hong Kong-impact of diabetes and cardiovascular disease status.** *Perit Dial Int* 2014, **34:**390-398.

**Legends to figures and tables**

**Figure 1** Follow chart for study participant enrollment and outcomes.

Note: PD peritoneal dialysis, HD hemodialysis, CVD cardiovascular disease.

**Figure 2** Cumulative incidence function curves for the cumulative incidence of CVD mortality (a) and all-cause mortality (b) in patients categorized by TyG-BMI quartiles.

**Figure 3** Subgroup analyses. A comparison of the adjusted hazard ratios of CVD mortality (a) and all-cause mortality (b) for the subgroups is presented by forest plot. Adjusted for age, sex, history of CVD, hemoglobin, albumin, LDL-C, urine output, and lipid-lowering agents for each subgroup (excluding for its own group)

**Table 1** Baseline characteristics of different TyG-BMI groups.

**Table 2** Association between TyG-BMI and reference parameters.

**Table 3** Association between TyG-BMI and CVD/all-cause mortality.

**Table 1.** Baseline characteristics of different TyG-BMI groups.

| **Characteristics** | **Total**  **(n=2,335)** | **Quartiles of TyG-BMI** | | | | | ***P* value** |
| --- | --- | --- | --- | --- | --- | --- | --- |
|  |  | **Q1 (n=584)**  **<165.5** | **Q2 (n=584)**  **165.5-<183.7** | | **Q3 (n=584)**  **183.7-<209.2** | **Q4 (n=583)**  **>209.2** |  |
| Age (years) | 46.1±14.8 | 40.4±15.1 | 45.2±15.1 | | 46.7±13.8 | 50.2±13.3 | <0.001* |
| Male sex (*n,* %) | 1382 (59.2) | 296 (50.7) | 357 (61.1) | | 376 (64.4) | 353 (60.5) | <0.001* |
| Dialysis duration (months) | 46.6 (22.4-78.0) | 48.8 (21.8-89.0) | 46.0 (21.6-76.2) | | 47.9 (24.4-75.3) | 43.5 (21.9-70.7) | 0.076 |
| Primary renal diseases |  |  |  | |  |  |  |
| Glomerulonephritis (n, %) | 1432 (61.3) | 461 (78.9) | 395 (67.6) | | 325 (55.7) | 251 (43.1) | <0.001* |
| Diabetic nephropathy (n, %) | 480 (20.6) | 33 (5.7) | 78 (13.4) | | 143 (24.5) | 226 (38.8) | <0.001* |
| Renal vascular diseases (n, %) | 179 (7.7) | 23 (3.9) | 43 (7.4) | | 51 (8.7) | 62 (10.6) | <0.001* |
| Others (n, %) | 244 (10.4) | 67 (11.5) | 68 (11.6) | | 65 (11.1) | 44 (7.5) | 0.069 |
| BMI (kg/m^2^) | 21.8±3.2 | 18.4±1.5 | 20.5±1.3 | | 22.5±1.4 | 25.6±2.6 | <0.001* |
| Systolic pressure (mmHg) | 136±20 | 135±19 | 137±20 | | 136±20 | 136±21 | 0.617 |
| Diastolic pressure (mmHg) | 85±14 | 87±13 | 86±14 | | 84±14 | 83±13 | <0.001* |
| Urine output (ml) | 1000 (600-1500) | 900 (500-1400) | 1000 (600-1500) | | 1000 (700-1600) | 1100 (600-1700) | <0.001* |
| **Comorbidities** |  |  |  | |  |  |  |
| Diabetes (*n,* %) | 564 (24.2) | 41 (7.0) | 96 (16.4) | | 170 (29.1) | 257 (44.1) | <0.001* |
| CVD history (*n,* %) | 782 (33.5) | 136 (23.3) | 181 (31.0) | | 213 (36.5) | 252 (43.2) | <0.001* |
| Hypertension (*n,* %) | 664 (28.4) | 79 (13.5) | 155 (26.5) | | 188 (32.2) | 242 (41.5) | <0.001* |
| **Laboratory parameters** |  |  |  | |  |  |  |
| Hemoglobin (g/l) | 101±22 | 101±24 | 101±22 | | 101±22 | 101±20 | 0.900 |
| Albumin (g/l) | 36.1±5.1 | 36.9±5.3 | 36.7±4.9 | | 36.5±5.1 | 36.6±5.1 | 0.593 |
| Triglyceride (mg/dl) | 124 (89-178) | 91 (68-122) | | 116 (89-156) | 130 (98-172) | 186 (134-274) | <0.001* |
| FBG (mg/dl) | 84.6 (75.6-100.8) | 79.2 (70.2-86.4) | | 84.6 (75.6-95.4) | 88.2 (77.4-104.4) | 95.4 (82.8-138.6) | <0.001* |
| TyG index | 8.62 (8.23-9.08) | 8.17 (7.88-8.49) | 8.54 (8.23-8.85) | | 8.69 (8.38-9.04) | 9.20 (8.78-9.65) | <0.001* |
| Total cholesterol (mg/dl) | 193±52 | 185±49 | 192±50 | | 196±50 | 202±55 | <0.001* |
| HDL-C (mg/dl) | 47.5±16.3 | 54.4±18.2 | 49.2±16.5 | | 45.7±13.5 | 40.8±13.2 | <0.001* |
| LDL-C (mg/dl) | 114±39 | 108±35 | 115±37 | | 119±39 | 116±42 | <0.001* |
| Serum creatinine (mg/dl) | 8.74±3.10 | 8.82±3.14 | 8.84±3.20 | | 8.64±2.83 | 8.68±3.23 | 0.661 |
| Uric acid (mg/dl) | 7.15±1.63 | 7.03±1.60 | 7.17±1.64 | | 7.07±1.62 | 7.32±1.66 | 0.009* |
| Urea nitrogen (mg/dl) | 49.8±29.2 | 50.7±21.0 | 49.2±20.4 | | 50.6±20.2 | 48.9±19.2 | 0.240 |
| eGFR (ml/min/1.73 m^2^) | 6.73±2.84 | 6.75±2.71 | 6.73±2.87 | | 6.70±2.65 | 6.73±3.12 | 0.926 |
| hs-CRP (mg/L) | 1.69 (0.66-5.31) | 0.86 (0.36-2.78) | 1.25 (0.24-3.95) | | 1.58 (0.57-4.84) | 2.92 (1.03-7.43) | <0.001* |
| **Medicine** |  |  |  | |  |  |  |
| Antihypertension agents, n (%) | 2010 (86.1) | 483 (82.7) | 498 (85.3) | | 509 (87.2) | 520(89.2) | 0.041* |
| Antidiabetics agents, n (%) | 331 (14.2) | 23 (3.9) | 48 (8.2) | | 96 (16.4) | 164(28.1) | <0.001* |
| Lipid-lowering agents, n (%) | 251 (10.7) | 29 (5.0) | 46 (7.9) | | 70 (12.0) | 106(18.2) | <0.001* |

BMI body mass index, CVD cardiovascular disease, FBG fasting blood glucose, TyG triglyceride glucose, HDL-C high density lipoprotein cholesterol, LDL-C low density lipoprotein cholesterol, eGFR estimated glomerular filtration rate, hs-CRP high-sensitivity C-reactive protein

**Table 2** Association between TyG-BMI and reference parameters.

| **Variables** | **Unstandardized regression coefficient** | | | **Standardized regression coefficient** | | ***T*** | ***P* value** |
| --- | --- | --- | --- | --- | --- | --- | --- |
|  | ***B*** | | **Standard error** |  |  | |  |
| Age (years) | 0.283 | | 0.056 | 0.122 | 5.063 | | <0.001* |
| Sex  (male *vs.* female) | -4.114 | | 1.462 | -0.059 | -2.815 | | 0.005* |
| History of CVD  (yes *vs.* no) | 4.595 | | 1.573 | 0.064 | 2.921 | | 0.005* |
| History of diabetes  (yes *vs*. no) | 21.058 | | 1.906 | 0.263 | 11.049 | | <0.001* |
| Systolic pressure (mmHg) | -0.036 | | 0.035 | -0.021 | -1.035 | | 0.301 |
| Hemoglobin (g/L) | -0.103 | | 0.035 | -0.065 | -2.929 | | 0.003* |
| Albumin (g/L) | 0.831 | | 0.154 | 0.124 | 5.386 | | <0.001* |
| LDL-C (mg/dl) | 3.119 | | 0.708 | 0.092 | 4.403 | | <0.001* |
| Urine output (ml) | | 0.008 | 0.001 | 0.141 | 6.806 | | <0.001* |
| Lipid-lowering agents  (yes *vs.* no) | | 10.304 | 2.169 | 0.099 | 4.750 | | <0.001* |

CVD cardiovascular disease, LDL-C low density lipoprotein cholesterol

**Table 3.** Association between TyG-BMI and CVD/all-cause mortality.

|  | **Model 1** | | **Model 2** | | **Model 3** | |
| --- | --- | --- | --- | --- | --- | --- |
|  | **HR (95% CI)** | ***P* value** | **HR (95% CI)** | ***P* value** | **HR (95% CI)** | ***P* value** |
|  | **CVD mortality** | | | | | |
| TyG-BMI (per SD increase ) | 1.38(1.24-1.53) | <0.001* | 1.24(1.10-1.39) | <0.001* | 1.28(1.13-1.45) | <0.001* |
| Quartile 2 | 1.47 (1.03-2.10) | 0.036* | 0.97 (0.68-1.40) | 0.883 | 0.93 (0.62-1.37) | 0.700 |
| Quartile 3 | 1.40 (0.98-2.00) | 0.068 | 0.83 (0.58-1.20) | 0.323 | 0.80 (0.54-1.20) | 0.280 |
| Quartile 4 | 2.46 (1.77-3.42) | <0.001* | 1.50 (1.08-2.10) | 0.016* | 1.51 (1.05-2.17) | 0.027* |
| **All-cause mortality** | | | | | | |
| TyG-BMI (per SD increase ) | 1.28(1.19-1.38) | <0.001* | 1.21(1.03-1.22) | 0.008* | 1.19(1.09-1.31) | <0.001* |
| Quartile 2 | 1.32 (1.04-1.69) | 0.023* | 0.92 (0.72-1.18) | 0.501 | 0.91 (0.69-1.19) | 0.487 |
| Quartile 3 | 1.42 (1.12-1.80) | 0.004* | 0.85 (0.67-1.08) | 0.194 | 0.91 (0.70-1.19) | 0.503 |
| Quartile 4 | 2.00 (1.59-2.51) | <0.001* | 1.23 (0.98-1.55) | 0.080 | 1.36 (1.05-1.75) | 0.018* |

HR, hazard ratio

CI, confidence interval

SD, standard deviation

Reference group was Quartile 1

Model 1: Unadjusted

Model 2: Adjusted for age, sex, systolic pressure, and history of CVD

Model 3: Adjusted for model 2 covariates and hemoglobin, serum albumin, LDL-C, urine output and use of lipid-lowering agents

**P* < 0.05
